# CD30+ lymphoproliferative disorder following immune checkpoint inhibition in a patient with Merkel cell carcinoma

**DOI:** 10.1016/j.jdcr.2025.04.035

**Published:** 2025-05-21

**Authors:** Aneri Bhargav Patel, William Driscoll, Luke Horton, Ajay Nair Sharma, Linda Doan, Janellen Smith

**Affiliations:** a Department of Dermatology, University of California, Irvine, California; b School of Medicine, University of California, Davis, California; c New York Genome Center, New York, New York

**Keywords:** immune checkpoint inhibitor, immunotherapy, lymphomatoid papulosis, Merkel cell carcinoma

## Introduction

Merkel cell carcinoma (MCC) is a rare cutaneous neuroendocrine malignancy that most often presents as an erythematous or indurated nodule or plaque in sun-damaged skin. The 5-year survival rate for local disease is about 51% and can be as low as 14% for metastatic disease.^1^ Patients with MCC have an increased risk of developing other lymphohematopoietic malignancies.

Lymphomatoid papulosis (LYP) is an indolent CD30-positive T-cell lymphoproliferative disorder (CD30+ LPD) that is thought to be part of the spectrum that includes anaplastic large cell lymphoma and borderline lesions. LYP is clinically characterized by widespread, recurrent, papulonodular, and necrotic lesions that, notably, unlike the malignant disorders of the CD30+ LPD spectrum, spontaneously regress. Patients with LYP are at increased risk of developing another hematologic malignancy such as large-cell CD30+ lymphoma, Hodgkin’s lymphoma, or mycosis fungoides.^2^ These can occur before, after, or simultaneously with the onset of LYP.^2^ Although there are 3 case reports of LYP due to immunotherapy,^3^, ^4^, ^5^ the coexistence of MCC and LYP has not been described, and this case highlights a unique confluence of pathologies.

## Case report

The patient presented to dermatology with a new erythematous nodule on the right elbow. Initial differential diagnoses included an inflamed cyst, for which an intralesional Kenalog injection was performed without improvement. A biopsy revealed atypical cells with stippled chromatin and scant cytoplasm infiltrating the dermis. Immunohistochemical studies demonstrated positive pankeratin with perinuclear dot staining and ISM1 positivity in a subset of cells consistent with MCC. Imaging showed an fluorodeoxyglucose (FDG)-avid skin lesion on the posterior-radial aspect of the elbow extending deep into subcutaneous tissues, with possible involvement of the underlying musculature and prominent right axillary lymphadenopathy suggestive of metastatic disease. Therefore, the patient was staged as IIIcT4N1 MCC on the right elbow.

**Fig 1:**
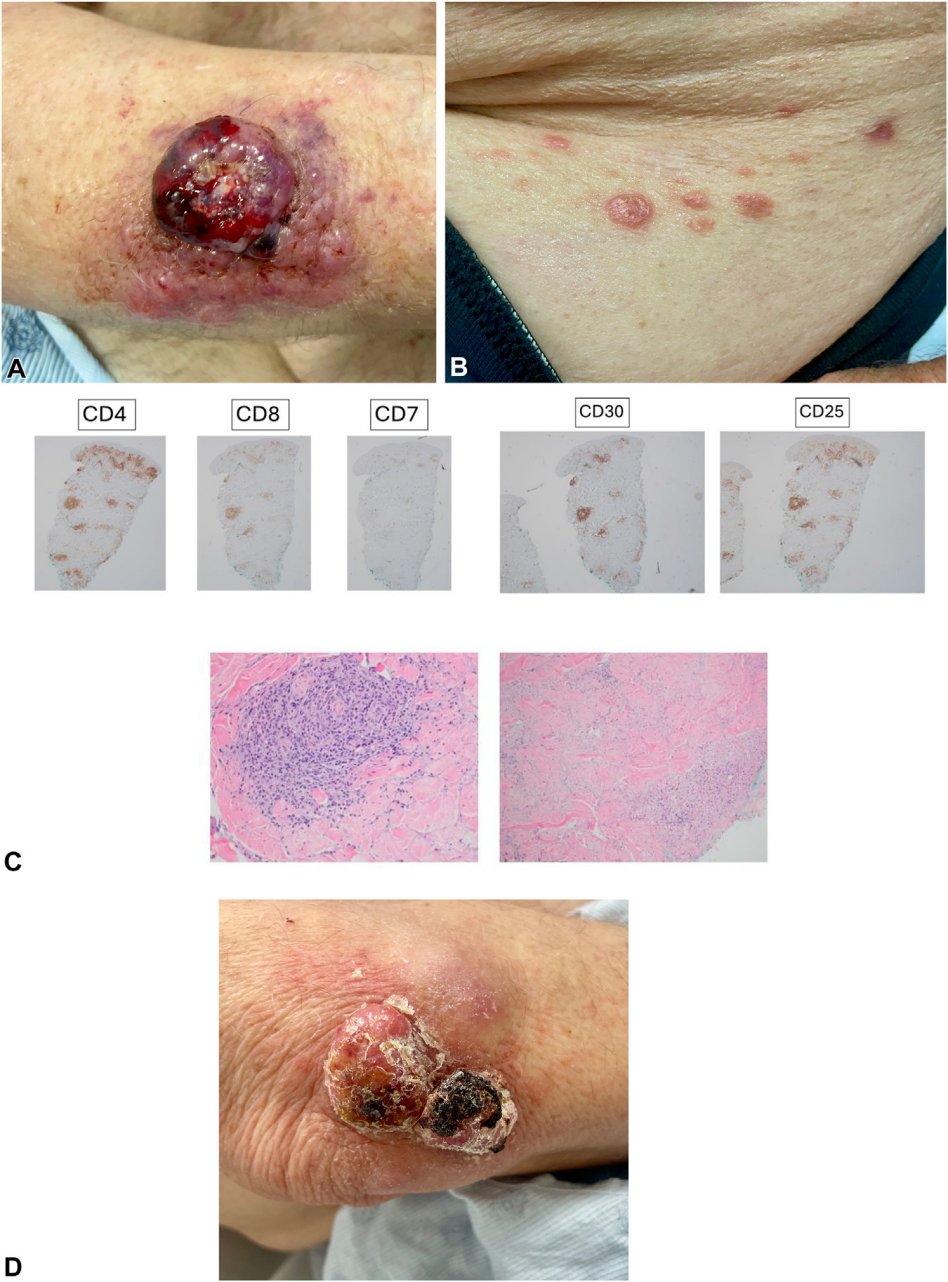
**A,** Exophytic and eroded plaque on right arm, consistent with MCC. **B,** Scattered relapsing and remitting erythematous dermal-appearing *pink-purple* papules on the right flank consistent with LYP. **C,** CD4, CD8, CD7, CD30, and CD25 stains of right abdominal lesion and hematoxylin and eosin (H&E) of right abdominal lesion. **D,** Eroded plaque on right arm, status postradiation treatment of MCC. *MCC*, Merkel cell carcinoma; *LYP*, lymphomatoid papulosis.

The patient’s lesion grew and became more exophytic, with erosion and erythema around the border (Fig 1, *A*). He was started on neoadjuvant nivolumab for MCC. Over the next few weeks, he developed new, small, raised, pink, pea-sized lesions that would often scale and crust and then resolve over a few weeks before recurring. Subsequent exams uncovered lesions on the left mandible and right abdomen (Fig 1, *B*). Biopsy of both the abdominal lesion and the mandibular nodule showed a dense perivascular infiltrate of atypical cells with high-grade features. Immunohistochemical studies revealed positivity for CD3, CD4, CD8, CD25, and CD30, with decreased CD7 (Fig 1, *C*). Given the self-resolving history of these lesions, along with the CD30+ immunophenotype, a diagnosis of LYP was made.

After 2 months of being on nivolumab, he was switched to avelumab due to the development of a diffuse rash, suspected to be a drug eruption. Concurrently, he received neoadjuvant radiotherapy to the right elbow, which resulted in a significant reduction of the primary tumor size (Fig 1, *D*).

As of this writing, the patient continues to experience relapsing-remitting lesions consistent with LYP, as well as new skin lesions that have been biopsy-proven to be MCC. His most recent positron emission tomography/computed tomography showed a mixed response to therapy, revealing new multifocal FDG-avid lesions on the right distal forearm that represented recurrent MCC, as well as new FDG-avid lesions on the left neck and right abdomen that correspond to the areas of recurrent LYP. Importantly, resolution of previously observed FDG-avid right elbow skin lesions and right axillary lymph nodes was noted, indicating a favorable response to radiotherapy and immunotherapy.

## Discussion

MCC is a rare primary malignancy that predominantly affects elderly Caucasian males. It is particularly prevalent among immunosuppressed individuals, including organ transplant recipients and those with HIV.^6^

LYP is an uncommon skin condition with an incidence of 1.2 to 1.9 cases per million people and an excellent 5- to 10-year survival rate ranging from 92% to 100%. Notably, patients with LYP are at an increased risk for developing secondary malignancies, including anaplastic large cell lymphoma (26.3%), Hodgkin lymphoma (3.5%), and mycosis fungoides (61.4%).^7^ These lymphomas are clonally related to LYP and can arise in 4% to 60% of affected patients.^7^

The pathophysiology of LYP is unclear, but studies suggest a genetic component, including abnormalities in CD30 transcription.^8^ Although the literature does not document any direct cases linking LYP to MCC, there have been 3 case reports of LYP and other CD30+ LPD developing following immunotherapy. Bush et al described a case of CD30+ infiltrate likely due to ipilimumab therapy in a patient with metastatic melanoma and chronic lymphocytic leukemia.^3^ Secondly, Morita and Kasai showcased a case of LYP in a patient on nivolumab for stage IV renal cell carcinoma.^4^ Lastly, Pattison et al reported a case of ulcerative LYP following treatment with nivolumab and brentuximab in a patient with recurrent Hodgkin lymphoma.^5^ These cases highlight a role of immunotherapy in the development of LYP. The mechanism of LYP development remains unclear: perhaps LYP is secondary to the underlying malignancy, or some propose that immunotherapy may reveal—or even initiate—a hidden predisposition for these T cells to multiply abnormally.^5^

Currently, there are no curative treatments for LYP; treatment is aimed at symptomatic management, with topical corticosteroids, methotrexate, and phototherapy used with variable success.^9^ For larger, symptomatic LYP lesions, surgical excision or radiotherapy may be considered. Our patient’s disease followed a relapsing-remitting course classic to LYP, and symptomatic treatment was provided with topical corticosteroids.

This case report expands the limited literature on LYP development following immunotherapy and reveals a potential association between MCC and LYP that has not been previously reported. Routine imaging (such as abdominal ultrasound/positron emission tomography) is not recommended for all patients that get diagnosed with LYP; instead, imaging should be reserved for cases with specific concerning clinical signs that may point toward a malignant or neoplastic process.

## Conflicts of interest

None disclosed.
